# Advances in 2,3-Dimethylmaleic Anhydride (DMMA)-Modified Nanocarriers in Drug Delivery Systems

**DOI:** 10.3390/pharmaceutics16060809

**Published:** 2024-06-14

**Authors:** Dong Wan, Yanan Wu, Yujun Liu, Yonghui Liu, Jie Pan

**Affiliations:** 1 School of Chemistry, Tiangong University, Tianjin 300387, China; wandong_tjpu@126.com (D.W.); 13611145467@163.com (Y.W.); 2 School of Chemical Engineering and Technology, Tiangong University, Tianjin 300387, China; liuyujun1021@126.com

**Keywords:** tumor microenvironment, 2,3-dimethylmaleic anhydride, pH responsive, drug delivery

## Abstract

Cancer represents a significant threat to human health. The cells and tissues within the microenvironment of solid tumors exhibit complex and abnormal properties in comparison to healthy tissues. The efficacy of nanomedicines is inhibited by the presence of substantial and complex physical barriers in the tumor tissue. The latest generation of intelligent drug delivery systems, particularly nanomedicines capable of charge reversal, have shown promise in addressing this issue. These systems can transform their charge from negative to positive upon reaching the tumor site, thereby enhancing tumor penetration via transcytosis and promoting cell internalization by interacting with the negatively charged cell membranes. The modification of nanocarriers with 2,3-dimethylmaleic anhydride (DMMA) and its derivatives, which are responsive to weak acid stimulation, represents a significant advance in the field of charge-reversal nanomedicines. This review provides a comprehensive examination of the recent insights into DMMA-modified nanocarriers in drug delivery systems, with a particular focus on their potential in targeted therapeutics. It also discusses the synthesis of DMMA derivatives and their role in charge reversal, shell detachment, size shift, and ligand reactivation mechanisms, offering the prospect of a tailored, next-generation therapeutic approach to overcome the diverse challenges associated with cancer therapy.

## 1. Introduction

Cancer and cardiovascular disease (CVD) are the leading causes of premature death in 127 countries. Current rankings and recent trends suggest that cancer may surpass CVD as the leading cause of premature death in most countries in this century [1]. Through decades of intensive research and considerable financial investment, researchers have made significant strides in understanding the causes and progression of cancer, which have greatly contributed to the development of cancer treatment strategies [2,3,4,5]. However, despite these advancements, a significant number of cancer patients still succumb to the disease each year. The National Cancer Centre (NCC) recently published the “Report on the Status of China’s Tumor Burden of Disease in 2022” via the Journal of the National Cancer Center (JNCC) [6]. It reveals that an estimated 4,824,700 new cancer cases are expected in 2022, with 2,533,900 cases in males and 2,290,800 in females. Additionally, approximately 2,574,200 cancer-related deaths are anticipated, with 1,629,300 in males and 944,900 in females [6]. These figures highlight the current challenges facing cancer treatment, which we can address through the development of novel chemotherapeutic agents. Drug efficacy can be improved by ameliorating the lack of selectivity and targeting of chemotherapeutic agents, which often lead to serious toxic side effects for patients [7]. Additionally, traditional chemotherapy agents are ineffective against drug-resistant tumor cells and fail to inhibit the metastasis of tumor cells [8,9]. The complex physiological environment within the body makes it difficult for conventional nanomedicine delivery systems to achieve precise treatment of tumors, as following entry into the cell, traditional drug delivery systems discharge chemotherapeutic agents into the cytoplasm, subsequently relying on the inefficient process of free diffusion to transport these agents into the nucleus [10,11,12,13,14,15,16]. Simultaneously, the intricate network of drug resistance mechanisms within tumor cells acts as a formidable barrier, effectively impeding the nuclear penetration of drug molecules [17,18,19]. A key example of such resistance is the action of P-glycoproteins, which are membrane-associated transporters capable of actively extruding drugs from the cellular interior [20,21].

Therefore, the ideal drug delivery system should not only have a stealth feature to extend its circulation time but also possess enhanced internalization capabilities at the tumor site [22,23,24]. Unfortunately, achieving both stealth and increased cellular uptake appears to be contradictory. Nanodrug delivery systems that are positively charged [25] or highly negatively charged [26] are rapidly cleared by the mononuclear phagocyte system (MPS) [7]. Consequently, in order to extend the duration of circulation within the bloodstream, the surface charge of nanomedicine delivery systems should be neutral or weakly negatively charged [27,28]. At the cellular level, however, positively charged nanomedicines facilitate enhanced cellular uptake through electrostatic interactions [29,30,31] and aid in the evasion of lysosomal degradation, thereby promoting drug delivery to target organs [17]. Integrating these two conflicting aspects into a single system is a complex yet gratifying task [32]. As a groundbreaking approach for alternative chemotherapeutic drugs, researchers are aiming to develop a “chameleon-like” drug delivery system that can enable prolonged blood circulation and enhance drug uptake by tumor cells within the body [33,34]. This innovative system is designed to possess a unique ability to remain dormant during blood circulation and become activated when targeting highly permeable tumor tissues through the enhanced permeability and retention (EPR) effect [9]. The concept of a “chameleon” drug delivery system implies that its internalization is turned off while in the bloodstream, but activated upon reaching the tumor cells. There is a self-contradictory relationship between stealth and cellular uptake in drug delivery [35]. Consequently, a larger number of nanomedicines can be successfully delivered to tumor cells, significantly improving the effectiveness of cancer treatment, and reducing toxic side effects [36,37].

**Figure 1:**
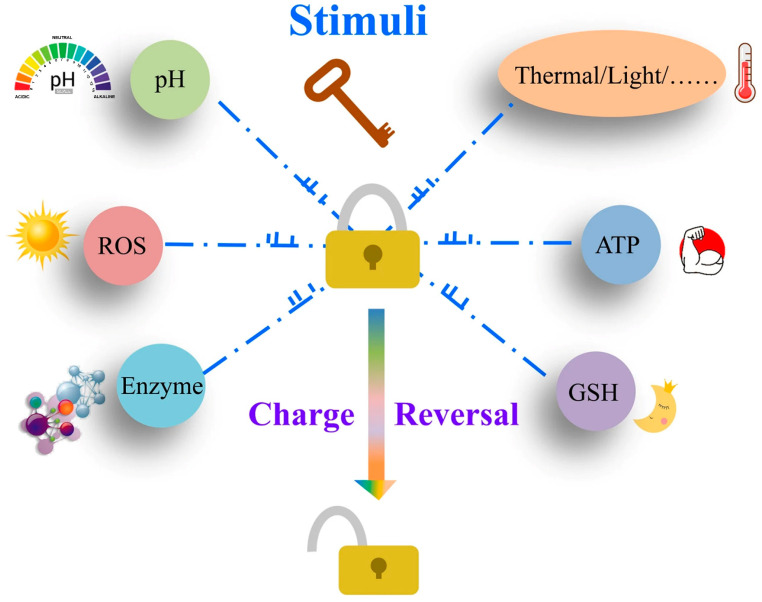
Illustration of the stimulus–response of charge-reversal carriers [58]. Reprinted with permission from [58]. Copyright 2020 BioMed Central.

The surface charge of nanocarriers has been found to play a crucial role both in vitro and in vivo. Research suggests that positively charged nanocarriers have a greater affinity for cell membranes with negative charged and are easily internalized by cells [38,39,40,41]. However, it is important to note that nanocarriers with positive charges may result in non-specific interactions with negatively charged serum components during blood circulation [42]. This can lead to the formation of blood clots in capillaries and potentially disrupt the structure of the plasma membrane, resulting in high cytotoxicity and an excessive immune response [43]. Conversely, negatively charged nanocarriers show promise in terms of protein resistance, which allows for prolonged circulation time in vivo [40,41]. This characteristic can be utilized in the design of innovative nanomedicine delivery systems that remain inert or invisible in the bloodstream but become more susceptible to recognition and uptake by tumor cells once they accumulate and activate at the tumor tissue site [44]. It is crucial to understand how the charge of nanocarriers can be adjusted through design to enhance drug uptake by tumor cells [32,45]. Charge-reversal nanocarriers maintain a negative charge under physiological conditions but can be induced to become positively charged by specific stimuli, such as changes in redox [46,47], pH [48,49,50,51], reactive oxygen species (ROS), enzymes [52,53,54,55,56], light, or temperature [57] (Figure 1). This switch facilitates prolonged blood circulation and enhanced uptake by tumor cells, thereby enhancing the efficacy of the delivered therapeutic agent against tumors [58].

In recent years, numerous researchers have dedicated their efforts to developing innovative drug delivery systems. Among these, stimuli-responsive drug delivery systems have garnered significant attention. These systems rely on various stimuli, such as pH difference, temperature difference, redox reaction, and enzyme response, to trigger drug release. Of these stimuli, pH responsiveness has been extensively studied due to the disparities between tumor tissues and normal tissues. Normal tissues have a neutral pH of around 7.4, whereas tumor tissues are weakly acidic, with a pH of approximately 6.8. Furthermore, tumor cells display an even more acidic pH of 4.5 to 6.3 in lysosomes and endosomes [59,60,61,62]. Currently, two approaches are commonly employed for a pH-stimulatory response. The first approach involves the usage of polymers as nanoparticle carriers with functional groups capable of altering charge density in response to pH changes [63,64]. The second approach entails incorporating breakable bonds into the nanoparticle carriers, allowing for the direct release of the drug molecules bound to or encapsulated in the carriers through bond-breaking [43,65,66,67,68,69].

The first type of charge-reversal strategy mainly relies on the protonation and deprotonation ability of polymer materials to achieve charge reversal in the tumor microenvironment through the difference in charge ratios in different acid environments [37,63,70,71]. However, this type of charge-reversal strategy has the obvious disadvantages of complicated operation process, difficult charge regulation, and the need for a deep understanding of the molecular structure of polymers and precise regulation ability [72,73,74]. The second type of charge inversion strategy entails the development of drug carriers modified with amide bonds, leveraging the pH differential between tumor and normal tissues [58]. In the tumor microenvironment, the amide bond is broken, exposing the original amine group, and leading to charge reversal through protonation and deprotonation. These acid-sensitive amide bonds exhibit stability under normal physiological conditions, thereby effectively extending the circulation time of the carrier in vivo. This process intensifies the interaction with the negatively charged tumor cell membrane and enhances tumor cell endocytosis. The acid anhydride-modified charge inversion system demonstrates substantial benefits compared to the first type of charge inversion strategies. Currently, the acid-sensitive chemical bonds commonly employed for achieving charge inversion include imine and β-carboxylic amide bonds, among others. β-carboxylic amide bonds can be further categorized into several types, such as maleic anhydride derivative amide bonds [75], citric acid amide bonds [76], cyclohexylenoic acid amide bonds of 1,2-dicarboxylic acids [77], and carboxydimethylenecarboxylic acid amide bonds [78]. Maleic anhydride derivatives are commonly used to develop intelligent drug delivery systems, and this review will specifically focus on the properties of maleic anhydride and its derivatives as well as the advancements in the field of drug delivery for cancer therapy based on DMMA-modified drug delivery systems [79].

## 2. Properties of Maleic Anhydride and Its Derivatives

With the rapid advancement of nanotechnology in cancer therapy, researchers have made strides in utilizing maleic anhydride derivatives in pH-responsive drug delivery systems [80]. This innovation takes advantage of pH changes in tumor tissues, effectively overcoming various biological barriers in vivo through the display of diverse charge characteristics in different tumor environments [21,81]. Traditionally, it was believed that common amide bonds are non-degradable or can only be broken down under extreme pH conditions [82]. However, recent findings have demonstrated unique characteristics of maleic anhydride derivatives [79]. Firstly, the pH sensitivity of maleic anhydride derivatives can be adjusted by introducing substituents to the cis-double bond. The presence of more substituents leads to increased sensitivity to acids. Secondly, the pH sensitivity of maleic anhydride derivatives with β-carboxylate groups is also influenced by charge density. Generally, maleic anhydride derivatives carry a negative charge under neutral or alkaline conditions due to the presence of carboxyl groups. However, when the amide bond degrades in an acidic environment, maleic anhydride derivatives transform into positively charged amine groups [83,84]. Therefore, the pH sensitivity of maleic anhydride derivatives can also be fine-tuned by adjusting the pH level.

Currently, numerous researchers are studying the development of charge-reversible drug delivery systems based on maleic anhydride derivatives [85]. These drug delivery systems utilize the pH differences found in different physiological sites [60]. When the drug carrier circulates in the blood or normal tissues, it exhibits negative electronegativity. This characteristic protects the drug carrier from being cleared by the blood and phagocytosed by normal cells. However, when the drug carrier reaches the tumor site or tumor cells, it undergoes a transformation into positive electronegativity. This positive charge increase enhances endocytosis of the cell on the drug carrier or promotes drug release [86]. This approach aims to achieve targeted drug delivery to the tumor [15,39,87]. The main maleic anhydrides used are 1,2-dicarboxycyclohexenoic anhydride (DCA), 2,3-dimethylmaleic anhydride (DMMA or DMA or DA), and 2,2,3,3-tetramethylsuccinic anhydride (TM) [66,78]. Researchers have focused on modifying nanomedicine drug delivery systems using different anhydrides [15,61,88]. Through this modification, nanomedicines have negatively charged surfaces that effectively prevent plasma protein coverage and mononuclear macrophage phagocytosis during blood circulation, significantly improving blood circulation time.

**Figure 2:**
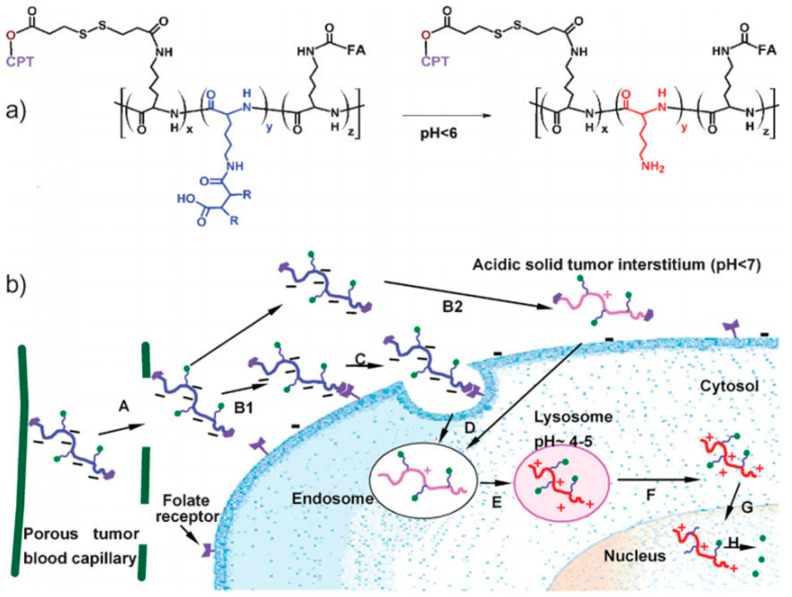
(**a**) Charge-reversible targeted PLL conjugated structure and its acid-triggered charge reversal; (**b**) Schematic diagram of drug delivery to the cell nucleus. The presence of the EPR effect causes these compounds to accumulate in the tumor tissue (A); a large number of folate receptors are present in the tumor tissue, and these folate receptors become the targeting points of the compounds, which are precisely localized and bound with the help of folate-targeting moieties (B1); they are endocytosed into the cell (C); translocation to the endosomes (D) followed by lysosomal uptake (E); the pH in the lysosome is about ~4–5, and this acidic environment favors the hydrolysis reaction of the amide, which in turn leads to PLL regeneration; the regenerated PLL carrier escapes from the lysosome into the cytoplasm (F) and diffuses into the nucleus (G), where the release of the drug it carries is accomplished. When PLLs are regenerated in the tumor tissue, they may adsorb to the cell membrane of the tumor cells, an adsorption process based on electrostatic interactions, which in turn triggers endocytosis (B2), allowing the drug to be taken up by the tumor cells more efficiently. Reprinted with permission from [89]. Copyright 2009 Wiley.

Zhou et al. demonstrated that the amide bonds formed from the reaction between these three anhydrides and amine groups have different sensitivities to acids [89] (Figure 2). Among them, DMMA was the most acid-sensitive, followed by DCA, and TM was the least sensitive. They also presented a drug delivery system that allows for charge reversal and targeting to the nucleus, resulting in enhanced drug cytotoxicity. By modifying polylysine (PLL) with DCA, the positive charge can be shielded by a potential amide, greatly reducing its interaction with plasma proteins during circulation in the bloodstream. Once the amidated PLL enters the cellular lysosome, the amide is hydrolyzed, regenerating the amine, and restoring the PLL’s ability to localize to the nucleus. At the same time, FA targeting groups are introduced on the surface, and the amidated PLL is modified with cleavable disulfide bonds, facilitating efficient drug uptake and delivery in tumor cells. However, the hydrolysis rate of this DCA-modified nanomedicine is slow in the acidic tumor microenvironment, hindering rapid charge reversal at the tumor site.

Since DMMA is highly sensitive to acidity compared to other maleic anhydride derivatives, it has been observed that the amide bond formed by the reaction of DMMA with amines can be cleaved in the tumor microenvironment [85]. Consequently, researchers have extensively utilized 2,3-dimethylmaleic anhydride, which can be cleaved under tumor acidity, as a key aspect for the development of pH-stimulated responsive drug delivery systems. This modified nanomedicine delivery system, with DMMA integrated, possesses a negatively charged surface during blood circulation, thereby preventing macrophage phagocytosis, and increasing the nanomedicine’s circulation time within the body. Furthermore, it can be hydrolyzed in the acidic tumor microenvironment to break the amide bond [90]. As a result, the nanomedicine undergoes changes in size and surface charge, promoting drug permeability and facilitating uptake and intracellular delivery by tumor cells. This property enables the overcoming of multiple biological barriers present in the organism, leading to improved drug delivery in vivo and enhanced therapeutic effects in cancer treatment [91]. Hence, the pH-sensitive degradation of maleic anhydride and its derivatives holds tremendous potential for the development of charge-reversible smart drug delivery systems.

## 3. Advances in DMMA-Modified Smart Nanomedicine Delivery Systems

**Figure 3:**
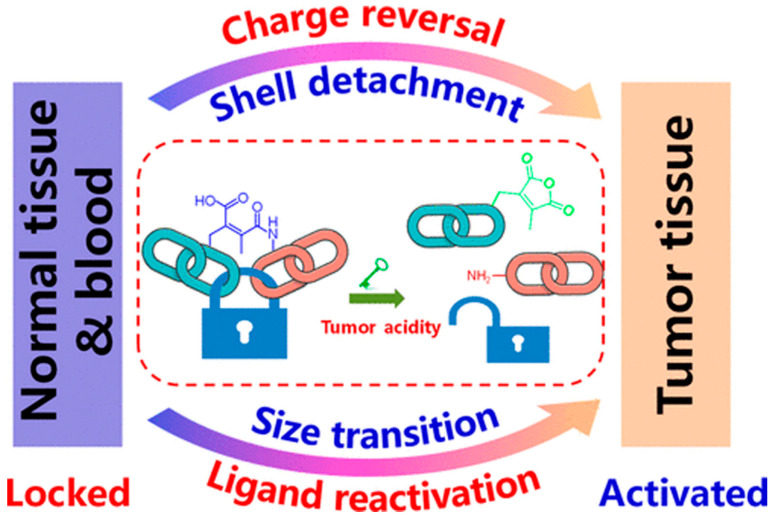
Hydrolysis of 2,3-dimethylmaleic acid amide bonds triggered by tumor acidity. Reprinted with permission from [79]. Copyright 2018 American Chemical Society.

In Figure 3, Wang et al. have developed an innovative drug delivery system using maleic anhydride derivative-modified weak acid-responsive drugs [79]. A notable feature of this drug delivery vehicle is the incorporation of DMMA and an amino-functionalized nanogel (PAMA) that react to form an amide bond, which can be cleaved under the acidic conditions found in tumor tissues. This reaction leads to a transition from a carboxylic-rich to an amino-rich group, resulting in a transformation from a negative to a positive charge on the surface. Consequently, more nanomedicines can be recognized and absorbed by tumor cells. This design significantly expands the application of maleic anhydride derivatives in the design of intelligent drug delivery systems, effectively overcoming the delivery challenges in cancer nanomedicine. Moreover, this strategy offers new insights and directions for the investigation of drug delivery systems that can be activated by the extracellular pH of tumors.

**Figure 4:**
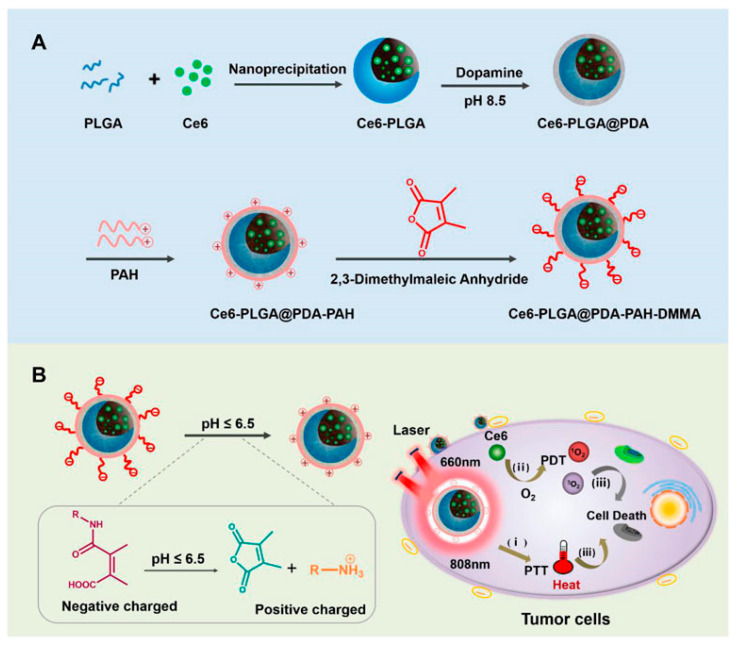
(**A**) Schematic of the preparation of a photothermal/photodynamic synergistic therapeutic nanomedicine delivery system; (**B**) Schematic of the in vivo circulation of nanomedicines [92]. Ce6-PLGA@PDA-PAH-DMMA NPs significantly enhance their endocytosis by cells through their surface-exposed positive charge properties, leading to efficient accumulation in tumor tissues. Once these nanoparticles are enriched at the tumor site, by applying 808 nm and 660 nm laser irradiation, these NPs are capable of rapidly generating large amounts of heat (photothermal effect) (i) as well as toxic reactive oxygen species (ROS) (ii). These two mechanisms work together to cause significant damage to the tumor tissue, ultimately effectively killing the tumor cells (iii). Reprinted with permission from [92]. Copyright 2022 Frontiers.

In Figure 4, Gao et al. have developed a nanodrug delivery system, designated Ce6-PLGA@PDA-PAH-DMMA, to potentiate cancer treatment through the augmentation of photothermal and photodynamic therapies’ synergistic effects [92]. The system features a DMMA-modified charge-reversal layer that allows nanocarriers to persist in the bloodstream under normal physiological conditions and to undergo charge reversal within the tumor microenvironment’s weak acidity, markedly enhancing their cellular uptake. In vitro studies have revealed that this nanomedicine system exhibits superior photothermal conversion capabilities and pronounced anti-tumorigenic properties, addressing the limitations inherent in conventional photothermal or photodynamic cancer therapies.

**Figure 5:**
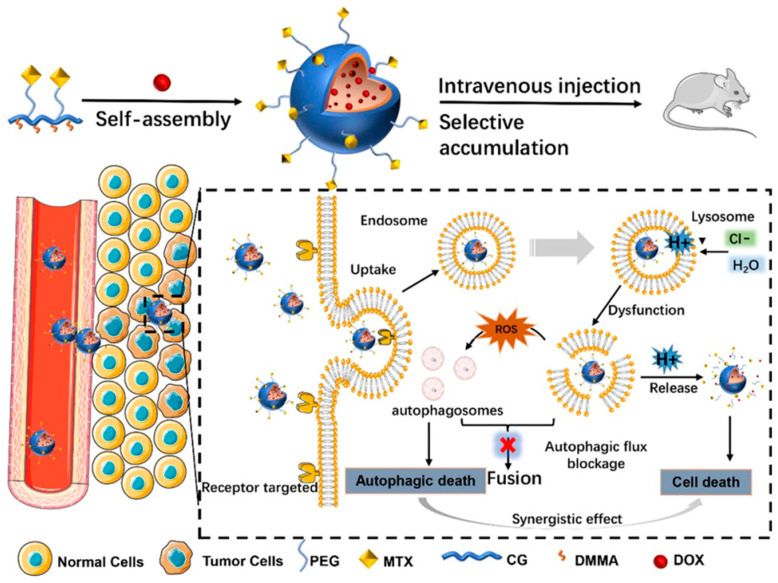
Schematic representation of micelles with autophagic flux interference [85]. Reprinted with permission from [85]. Copyright 2023 BioMed Central.

As shown in Figure 5, Cao and colleagues have successfully developed a novel drug delivery system, namely, methotrexate-polyethylene glycol (MTX-PEG) micelles modified with CG/DMMA [85]. In this system, a negatively charged polymer is formed by the conjugation of CG and DMMA, while the chemotherapeutic agent DOX is effectively encapsulated through electrostatic interactions. Within the acidic milieu of lysosomes, the amide bond between CG and DMMA undergoes cleavage, liberating the primary amine of CG and the carboxyl group of DMMA. These groups are capable of proton capture, triggering a substantial influx of chloride ions and water, resulting in lysosomal rupture. This approach not only enhances the tumor selectivity of DOX but also interferes with autophagic flux, substantially reducing systemic toxicity and markedly improving anti-tumor efficacy. In both in vitro and in vivo experiments, these micelles have exhibited superior anti-tumor activity and specificity, concurrently diminishing the accumulation and injury of chemotherapeutic drugs within healthy organs.

**Figure 6:**
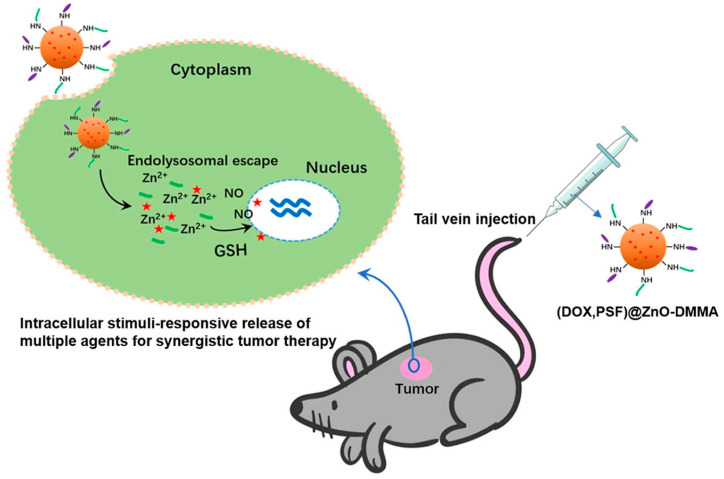
(DOX,PSF)@ZnO-DMMA nanoparticles synthesis strategy and schematic of intracellular drug delivery [93]. The red stars in this Figure are indicated the chemotherapy drug DOX. Reprinted with permission from [93]. Copyright 2020 Elsevier.

In Figure 6, Ding et al. synthesized a novel formulation of zinc oxide nanoparticles (ZnO NPs) functionalized with 2,3-dimethylmaleic anhydride (DMMA) and loaded with adriamycin (DOX) and phenylsulfonyl furoxan (PSF) [93]. The chemotherapeutic agent DOX and the nitric oxide (NO) precursor PSF were conjugated to the ZnO NPs via ligand and covalent bonds, respectively. Subsequently, DMMA modification of the nanoparticle surface induced a charge inversion, yielding (DOX, PSF)@ZnO-DMMA NPs. These multifunctional NPs could release Zn^2^⁺, DOX, and NO within cancer cells, aiming to elicit synergistic anti-cancer effects. Notably, NO release significantly enhanced the intracellular DOX concentration and mitigated multidrug resistance. The synergistic action of DOX and Zn^2^⁺ ions suppressed tumor growth. Moreover, the incorporation of DMMA prolonged the NPs’ circulation in the bloodstream, facilitated their accumulation at tumor sites, and augmented their uptake by cancer cells. In vitro and in vivo studies confirmed that these NPs effectively inhibited cancer cell proliferation and markedly reduced multidrug resistance (MDR).

**Figure 7:**
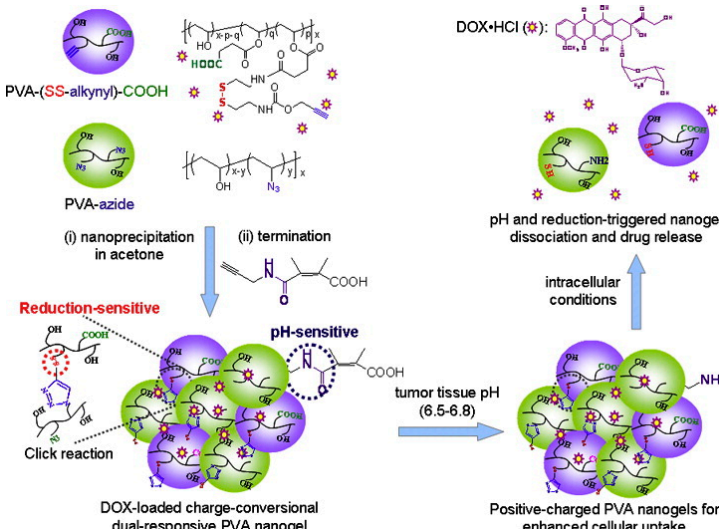
Illustration of charge conversion and reduction-sensitive PVA nanogels for enhanced cellular uptake and intracellular DOX release. Reprinted with permission from [69]. Copyright 2015 Elsevier.

In Figure 7, Chen et al. have developed an improved polyvinyl alcohol (PVA) nanogel that can enhance cellular uptake and trigger the release of DOX for more effective cancer treatment [69]. The researchers used reverse nanoprecipitation to prepare the PVA nanogel and introduced DMMA to improve the encapsulation rate by enabling electrostatic interactions. Moreover, the nanogel contains a super pH-sensitive end group that changes its surface charge from negative to positive in the slightly acidic extracellular pH found in tumors, promoting its uptake by cancer cells. Furthermore, the nanogel features disulfide bonds that can be reduced by intracellularly expressed homoglutathione, resulting in the rapid release of DOX for targeted and controlled intracellular drug delivery. This dual-responsive system can respond to the extracellular pH of tumors, as well as the endosomal pH and intracellular glutathione (GSH) levels, which ensures enhanced cellular uptake and more efficient intracellular drug release.

**Figure 8:**
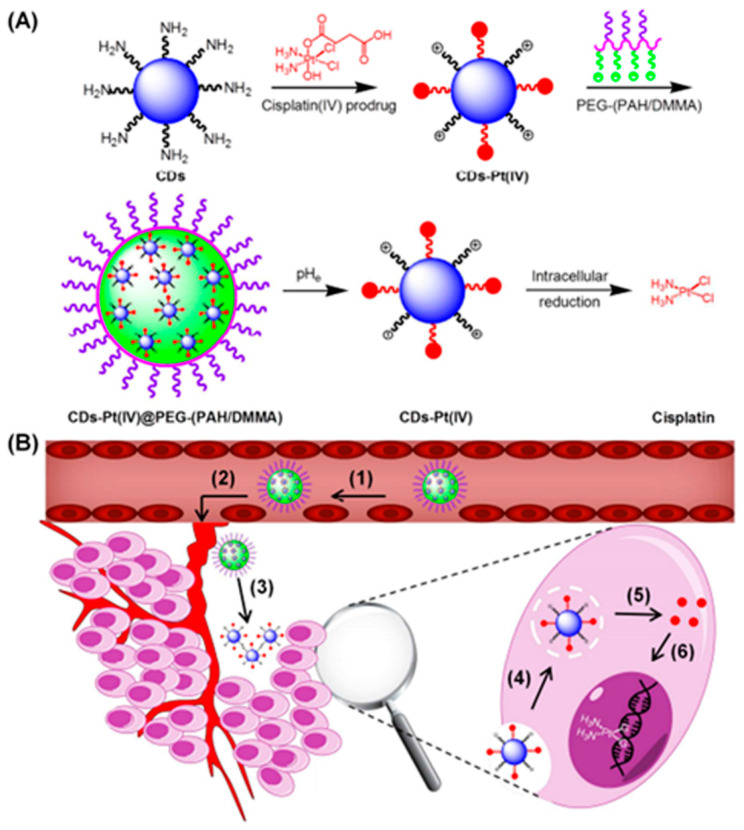
(**A**) Schematic diagram of the preparation of charge convertible CDs-based drug delivery system; (**B**) Schematic diagram of the drug delivery process. Reprinted with permission from [94]. Copyright 2016 American Chemical Society.

Feng et al., in Figure 8, fabricated a novel tumor extracellular microenvironment-responsive drug delivery system using cisplatin (IV) prodrug-loaded charge-convertible cyclodextrins (CDs) for imaging-guided drug delivery [94]. In the weakly acidic tumor extracellular microenvironment (pH ~6.8), anionic polymers with DMMA modification can transform into cationic polymers through charge-reversal transformation, leading to strong electrostatic repulsion and subsequently releasing positively charged nanocarriers, CDs-Pt (IV). Importantly, the positively charged nanomedicine delivery system exhibited a strong affinity for negatively charged cancer cell membranes, thereby enhancing the uptake of nanomedicines by cancer cells. This charge-convertible drug delivery system demonstrated superior therapeutic efficacy in the extracellular microenvironment of tumors compared to normal physiological conditions and non-charge-convertible nanocarriers. Additionally, the charge-convertible CDs exhibited high tumor suppression effects while showing low toxicity, highlighting their potential to enhance the therapeutic effect of smart drug nanocarriers.

**Figure 9:**
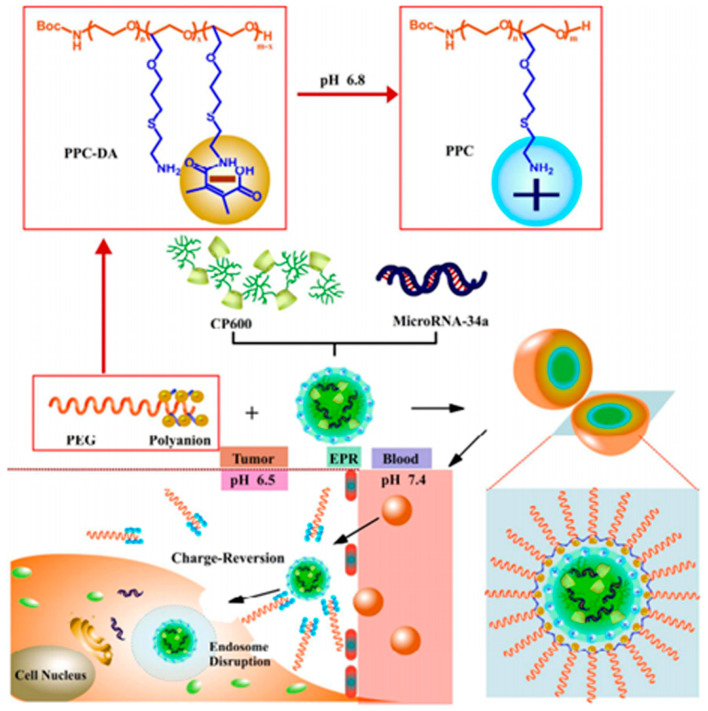
Schematic structure and mechanism of acid-responsive ternary nanocarriers with detachable shells. Reprinted with permission from [95]. Copyright 2017 American Chemical Society.

Fan et al. have successfully developed ternary nanocarriers with detachable shells for tumor-specific delivery of microRNA [95] (Figure 9). The nanocarriers are loaded with miR-34a and have a DMMA-modified polyethylene PEG anionic polymer coating. The synthesis of these nanocarriers involves the assembly of positively charged cationic polymers with microRNA-34a through electrostatic interactions. Subsequently, anionic PEG derivatives are coated onto the nanocarriers through electrostatic interactions in an acidic tumor microenvironment. This double-layered coating results in nanocarriers with a nearly neutral surface charge, minimizing non-specific adsorption and enabling them to circulate undetected by the immune system. Once within the acidic tumor microenvironment, the PEG shell of the nanocarriers is shed, exposing a positive charge. This positive charge facilitates uptake of the nanocarriers by tumor cells and promotes their escape from lysosomes, greatly enhancing the intracellular delivery and bioavailability of the drug. Furthermore, the shedding of the PEG shell also triggers the release of microRNA-34a, leading to downregulation of CD44 expression and inhibition of tumor growth.

**Figure 10:**
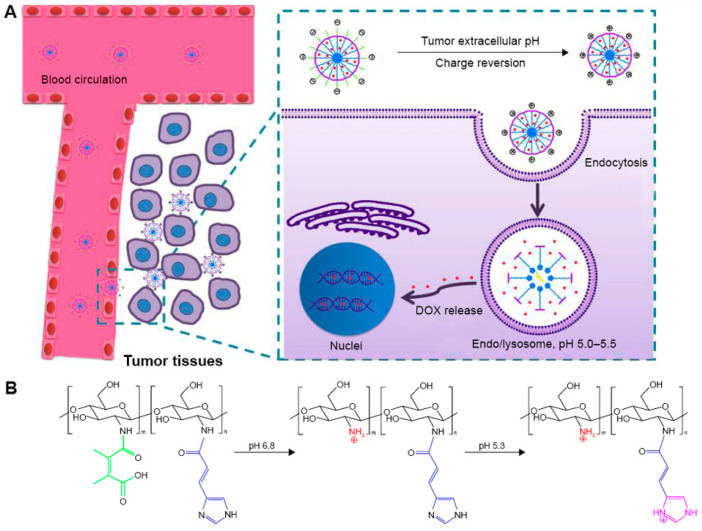
(**A**) DOX delivery process with stepwise pH-responsive NPs; (**B**) pH responsiveness of the polymer Da-cs-Ua [96]. Reprinted with permission from [96]. Copyright 2016 Dovepress.

Chen et al. have developed a novel pH-responsive nanomedicine delivery system that enables efficient delivery of DOX and contributes significantly to its therapeutic effect [96] (Figure 10). This system comprises 2,3-dimethylmaleic acid-chitosan-urocanic acid (DA-CS-UA), which triggers a stepwise response to extracellular and intracellular pH for therapeutic purposes. The nanocarriers (NPs) possess a negative surface charge and appropriate nano-size under physiological conditions. They also exhibit low non-specific adsorption to serum proteins and demonstrate favorable stability during blood circulation. When this nanomedicine delivery system enhances accumulation at the tumor site through the EPR effects, a two-step response occurs, leading to the exertion of drug efficacy. The first step involves a pH response that significantly promotes tumor cell uptake of DOX-loaded NPs. In this step, the slightly acidic tumor extracellular environment triggers DMMA hydrolysis, causing a negative-to-positive surface charge reversal of the NPs. Once the nanomedicine delivery system is internalized by the tumor cell, the second step involves a pH response in the endo/lysosomal acidic environment. This response leads to on-demand intracellular release of DOX from the NPs, enhancing cytotoxicity to the tumor cell. This step-by-step pH response to NPs demonstrates enhanced antiproliferative effects and reduced systemic side effects in vivo.

**Figure 11:**
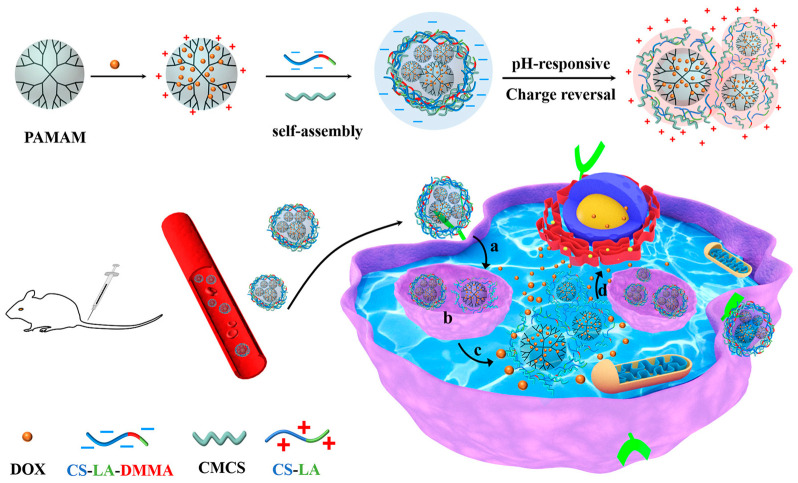
Schematic illustration of intracellular drug release mechanism and lysosomal escape of nanopreparations [97]. Reprinted with permission from [97]. Copyright 2021 Elsevier.

Sun et al. in Figure 11 developed a novel polysaccharide-based core-shell structured nanomedicine delivery system aimed at overcoming biological barriers within the blood circulation and lysosomes that frequently impede the efficacy of nanomedicines [97]. This system markedly augmented the anti-tumor effect of the chemotherapeutic agent doxorubicin (DOX) via a pH-triggered charge-reversal mechanism. The core-shell nano-formulation employs carboxymethyl chitosan (CMCS) as a bridge molecule, a negatively charged chitosan derivative (CS-LA-DMMA) as the outer shell, and integrates it with a positively charged PAMAM@DOX core. This design extends the circulation time of the nano-formulations in vivo and facilitates the positively charged core’s effective escape from the lysosome under pH alterations, through the ‘proton sponge effect’ and cation-anion interactions with the lysosomal membranes. This enhances the stability of the nanodrug and its sensitivity to pH changes. In vitro results revealed that the nano-formulation was efficiently internalized by tumor cells and induced significant apoptosis. Furthermore, the high accumulation of the nano-formulation in tumor tissue further augmented its ability to inhibit tumor growth.

**Figure 12:**
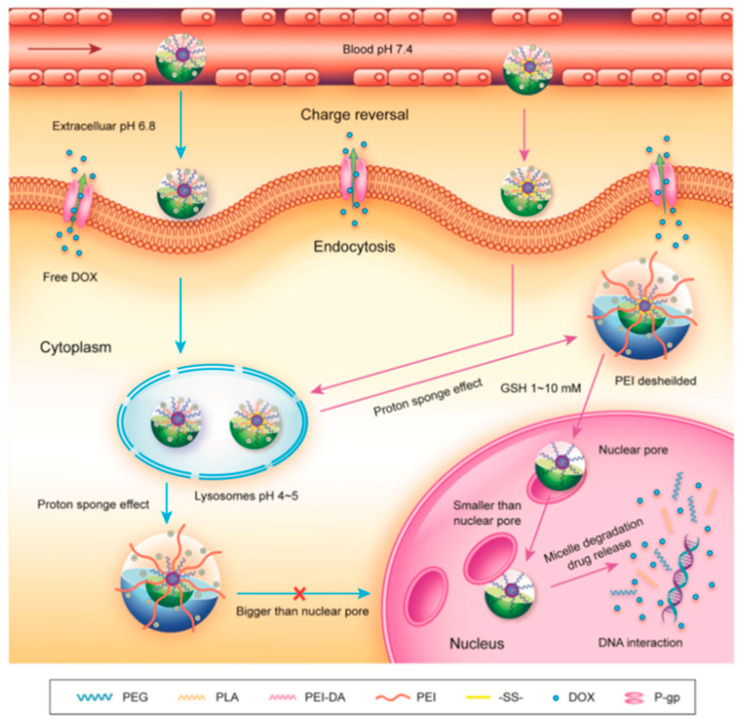
Schematic process of PELE-ss-DA micelles loaded with DOX for MDR cancer therapy. Reprinted with permission from [98]. Copyright 2015 Wiley.

Guo et al. introduced a groundbreaking polymeric micellar system that allows for size variation, enabling the direct delivery of an anticancer drug to the nucleus of MDR tumor cells [98] (Figure 12). These micelles feature a well-defined nucleus–crown structure, with poly(propylene lactone) (PLA) chain segments serving as the core material due to their exceptional biocompatibility and biodegradability. They are connected to methoxy poly(ethylene glycol) (mPEG) at one end and poly(ethylene imine) (PEI) at the other end through disulfide bonds. To ensure prolonged blood circulation and excellent stability under physiological conditions (pH 7.4), the micelles conceal their positive charge via the amination of PEI. Subsequently, the micelles accumulate in the tumor tissue through the EPR effect, facilitating internalization by tumor cells through attractive electrostatic interactions induced by the tumor tissue’s acidic pH. As the micelles reach the lysosome, they escape through the proton sponge effect of PEI, thus releasing them into the cytoplasm. Furthermore, the disulfide bond between PLA and PEI breaks in response to intracellular GSH, resulting in the shedding of the PEI shell and the transformation of the micelles into smaller particles that can pass through the nuclear pore and deliver the drug within the nucleus. In contrast, reduction-insensitive mPEG-PLA-PEI-DMMA (PELE-DA) micelles do not shed the PEI shell, limiting access to the nucleus due to their larger particle size.

## 4. Conclusions

Among various methods of pH responsiveness, nanocarriers targeted at the acidic microenvironment of tumor tissues represent a novel approach for tumor-specific drug delivery. Compared to traditional methods of achieving drug delivery through surface specificity, nanocarriers that target the acidic microenvironment in solid tumors seem to have a wider range of applications. Consequently, strategies focusing on the extracellular pH environment of tumors are widely adopted. In particular, the use of DMMA modification on nanocarriers has garnered significant interest among researchers. This modification enables the nanomedicine delivery system to acquire a negative surface charge during blood circulation, preventing interference from plasma proteins and mononuclear macrophages, thereby extending its circulation time. This extended circulation time facilitates the nanomedicine’s accumulation in tumor tissues through the EPR effect. However, once these nanomedicines accumulate at the tumor site, they undergo DMMA hydrolysis in the weak acid tumor tissue environment. This hydrolysis triggers a charge reversal of the nanocarriers through protonation and deprotonation, converting the nanomedicines’ negatively charged surface to a positively charged surface. As a result, more nanomedicines are taken up by tumor cells through electrostatic interactions. This significantly enhances the killing effect of nanomedicines on tumor cells while minimizing toxic side effects on normal cells.

## 5. Outlook

With the backdrop of advancements in cancer molecular biology, fourth-generation anticancer drugs—molecularly targeted therapies—have garnered significant interest. These therapies are designed to interfere with key target molecules involved in carcinogenesis or tumor growth, thereby reducing the toxic side effects of chemotherapeutic drugs on cancer patients. However, it is worth noting that molecularly targeted therapies only show efficacy in approximately 30% of patients, and the presence of genetic differences means that other patients may not receive the same benefits. Therefore, there is an urgent need to develop more precise and personalized medicines to better meet the multifaceted needs of clinical care.

After over a decade of extensive research, significant progress has been achieved in the development of tumor microenvironment-responsive nanocarriers as intelligent drug delivery systems. One such cutting-edge system is the pH stimulation-responsive drug delivery system, which has emerged in recent years. Previous studies have established that nanomedicines with negatively charged surfaces exhibit prolonged circulation in the blood, while positively charged nanomedicines are more readily internalized by cells compared to neutral and negatively charged counterparts [99]. In light of this, it becomes imperative to comprehend how nanoparticle charge can be purposefully modified to augment the uptake of nanomedicines by tumor cells [100]. This review extensively highlights the immense potential of pH-stimulated responding nanomedicine delivery systems, modified by DMMA, in the realm of cancer therapy. By doing so, it paves the way for novel opportunities and ideas to enhance the effectiveness of tumor-targeted therapies.

## Data Availability

Data are available on request.
